# Application of Remote Power-by-Light Switching in a Simplified BOTDA Sensor Network

**DOI:** 10.3390/s131217434

**Published:** 2013-12-17

**Authors:** Mikel Bravo, Angel Ullan, Ander Zornoza, Alayn Loayssa, Manuel Lopez-Amo, Jose Miguel Lopez-Higuera

**Affiliations:** 1 Department of Electric and Electronic Engineering, Universidad Pública de Navarra, Pamplona 31006, Spain; E-Mails: azorno3@uic.edu (A.Z.); alayn.loayssa@unavarra.es (A.L.); mla@unavarra.es (M.L.-A.); 2 Department TEISA, Universidad de Cantabria, av. Los Castros s/n, Santander, Cantabria, Spain; E-Mails: aun1979@hotmail.com (A.U.); lopezhjm@unican.es (J.M.L-H.)

**Keywords:** BOTDA sensor, remote sensing, optical switch, sensor network

## Abstract

We propose and demonstrate the use of spatial multiplexing as a means to reduce the costs of distributed sensing networks. We propose a new scheme in which remote power-by-light switching is deployed to scan multiple branches of a distributed sensing network based on Brillouin Optical Time Domain Analysis (BOTDA) sensors. A proof-of-concept system is assembled with two 5-km sensor fiber branches that are alternatively monitored using a fast remotely controlled and optically powered optical switch. The multiplexed distributed sensor fibers were located 10 km away from the interrogation unit and a Raman pump is used to remotely power the switch. Furthermore, the deployed BOTDA unit uses an alternative configuration that can lead to simplified setups.

## 1. Introduction

Optical fiber sensors are nowadays a good alternative for structure health monitoring. Fiber Bragg Grating (FBG) based sensors and fiber distributed based sensors have been the most commonly implemented techniques up to now [1]. Among distributed ones, Brillouin Optical Time Domain Analysis (BOTDA) systems are especially interesting since they are able to measure temperature and/or strain in a distributed manner all along the structure. The spatial resolution of commercial systems is typically limited to 1 m due to the phonon lifetime [2]. However, a number of novel techniques have been recently devised to attain sub-meter spatial resolutions [3–5]. Moreover, long range BOTDA systems have also been presented, attaining measurement of tens of kilometers or even longer distances when they are combined with Raman amplification along the channel [6]. Nevertheless, the widespread application of BOTDA technology is currently being hindered by the cost and the complexity of the setups that are necessary.

Multiplexing sensors is the most reasonable technique in order to reduce the cost of operation and increase the performance of a monitoring network. In these schemes, different kind of sensors can also be combined and multiplexed, enhancing the sensing capabilities of the system [7]. The use of optical switches in spatial multiplexing schemes can be crucial for reducing the cost of structural health monitoring, since they allow sensing multiple areas of large structures by means of a single interrogation unit that does not need to be capable of long length measurements. Moreover, they can also be used to enhance the robustness of the network against fiber breaks by implementing protection schemes. Ideally, in order to implement spatial multiplexing in the most cost-effective manner, these optical switches should be deployed in the field away from the interrogation unit. However, then the powering of these switches becomes a major issue that needs to be addressed in order to fulfill this vision. In this context, optical powering is a key technology for a new generation of intelligent sensor networks with a broad range of monitoring applications. Fiber optic power by light systems has been used since 1978 [8]. Optical powering is a key enabler for a new generation of autonomous multifunctional intelligent subscriber and sensor networks [9] with a broad range of monitoring and communication functions including home security and control of public spaces, roads, bridges and personal health, as well as to general-purpose communications, to name just a few. One can also envisage optical powering of short-range passive optical networks (PON) comprising distributed link-supervision [10]. Power by light optical switching was already demonstrated by Ogawa, who developed a multi-point optical sensing system presented in [11].

In this paper, we present a step in the development of sensor networks appropriate to monitor multiple structures with one single BOTDA system. A proof-of-concept system is demonstrated using a single power-by-light optical switch for selecting the structure to be sensed. This switch is controlled and powered remotely and optically, so no power supplies are needed away from the interrogation station. In addition, the BOTDA configuration used to interrogate the sensor network is simplified with respect to typical BOTDA setups. It uses a Brillouin generator using a highly nonlinear fiber as an optical frequency shifting device in the process of generation of the Brillouin pump and probe signals, hence the use of costly wideband electro-optic modulators (EOM) and microwave synthesized generators. This paper further extends preliminary work done by our group [12] with an enhanced explanation of the system concept, of the theoretical underpinnings of the Brillouin generator, detailed description of the BOTDA interrogation unit and additional experimental measurements.

## 2. Description of the System

**Figure 1.**
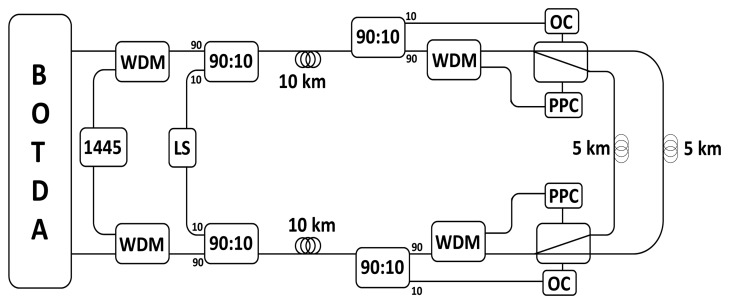
Structure of the BOTDA sensor network. WDM: wavelength division multiplexer; PPC: photoelectric cell; OC: optical control; LS: Laser Source; 1,445: 1,445 nm Raman pump.

The proposed sensor network is depicted in Figure 1. It consists of a BOTDA system used to interrogate a 25 km long fiber channel, where the central 5 km are connected through two optical switches, where the structures to be sensed and multiplexed are intended to be located. For controlling these switches different elements have to be added to the typical BOTDA network. The powered by light remote switches need, on one hand, a high power pump source at 1,445 nm to feed a photoelectric cell that supplies the power to the switches. On the other hand, a control signal is necessary to select the output port of the switch. A 1,445 nm Raman pump laser is used to feed the cell attached to the switches, and it is inserted into the network using two 1,445/1,550 nm WDMs. Another optical signal is introduced in the fiber lines to remotely control both switches position by two 90:10 coupler. These couplers are optimized to operate in the O (1,300 nm) and C (1,550 nm) bands, however, they do not introduce higher loss at 1,445 nm pump wavelength. Just before the switches, both signals are subtracted. The control signals are extracted by other two 90:10. The 10% signal for controlling the switch also has the 10% part of the Raman pump and Brillouin signal. Thus, this light is filtered by a circulator and an FBG centered at the control signal wavelength. Before arranging the control signal, the Raman pump necessary to feed the switch is removed by other two WDMs with the same characteristics as the previous ones.

**Figure 2.**
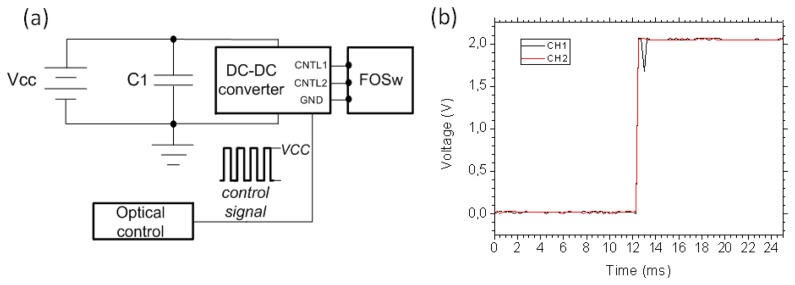
(**a**) Electronics setup for the remote control of the fiber optic switch; (**b**) Zoom of the switch rising flank response for both channels.

We used the photoelectric cell to drive a commercial, low consumption 1 × 2 fiber optic switch based on MEMS technology and developed by DiCon Fiberoptics Inc. (Richmond, VA, USA) A power converter, working at 1,445 nm, is used to convert 125 mW of optical power into ∼60 mW of electrical power. In order to select the channel, two different voltages are applied on the electrical ports of the switch. As the photoelectric cell provides a maximum of 4.8 V, DC-DC voltage converters are necessary to drive the optical switch as it is shown in Figure 2a. A remote optical control (OC) for the powered by light switch is developed. A laser source (LS) centered in 1,540 nm is used as the control signal. This light is inserted into the network by a 90:10 coupler and another 90:10 coupler is used to extract 10% of the power at the OC location. The control wavelength is filtered and photodetected. Finally, depending on the detected intensity, one of the two switch's channels is selected.

For measuring the switch response, the light coupled at the input port was detected and monitored by an oscilloscope. Figure 2b shows the rising flank of the switch response. A switching time less than 2 ms is observed.

### 2.1. Simplified BOTDA

**Figure 3.**
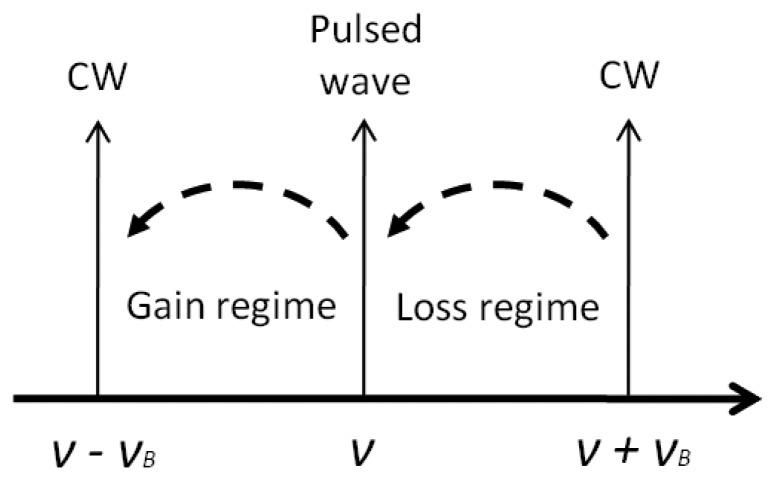
BOTDA signals relationship in gain and loss regime. The dashed line represents the energy transfer.

The BOTDA technique is based on the analysis of the Brillouin interaction between two counter propagating optical waves. One of them is a continuous wave (CW), the probe, while the other is pulsed, the pump. They have to be separated in frequency the value of the Brillouin frequency shift (*ν_B_*) for the fiber where the process takes place in order to induce stimulated Brillouin scattering (SBS). When this occurs, there is an energy transfer from one wave to the other, giving rise to a Brillouin spectrum that can be measured by tuning the frequency separation between pump and probe. The peak of this spectrum gives the Brillouin frequency shift in the fiber, which depends on temperature and strain. Moreover, using classic time-domain reflectometric techniques the distribution of these measurants along the fiber can be determined. There are two possible configurations for a BOTDA system: the so-called gain regime, when the pulsed wave is used as pump wave to amplify the CW (probe), and the so-called loss regime, when the CW is used to amplify the pulsed wave and is attenuated in the process. In both cases, the energy transfer occurs from the wave with higher frequency to the wave with lower frequency as shown in Figure 3.

**Figure 4.**
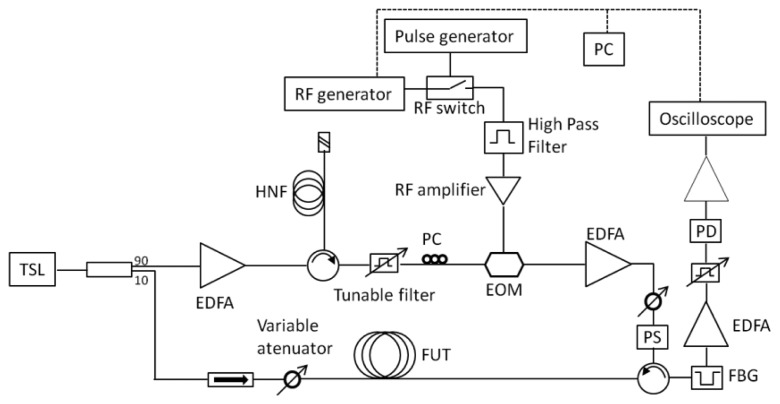
Scheme of the BOTDA setup used for our measurements. TSL: tunable semiconductor laser; PC: polarization controller; PD: photodetector; HNF: highly nonlinear fiber; PS: polarization scrambler; FUT: fiber under test.

The two signals, pump and probe, need to be locked to each other, so that the frequency gap remains constant, and this gap has to be tunable in order to scan the Brillouin spectra along the fiber. Usually, this is done by splitting in two parts a laser output and modulating one of them at the Brillouin frequency [13]. Another solution is to use two different laser sources locked in frequency or using the injection locking technique to generate the pump and probe signals with two DFB laser sources [14]. In every case, expensive components like wideband electro-optical modulators, microwave synthesized generators or wideband detectors are required. To interrogate our sensor network, we used a simplified BOTDA configuration, proposed by A. Zornoza *et al.* [15], that uses the Stokes wave generated inside a highly nonlinear fiber (HNF) to generate the probe wave. It operates in loss regime and it is depicted in Figure 4.

**Figure 5.**
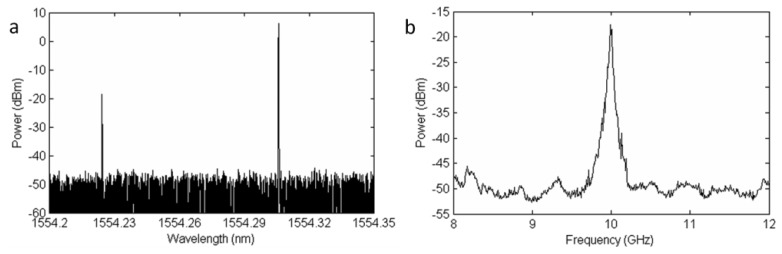
(**a**) Optical spectrum exiting the HNF; (**b**) Spectrum of the beat wave generated by the TLS output and its Brillouin scattering wave. Note that the peak is located at 9.992 GHz, in agreement with the 80.5 pm of separation measured in the optical domain.

The laser source is an external cavity tunable semiconductor laser (Santec TSL-210) centered at 1,554.23 nm with a linewidth of 1 MHz and whose output power (10 dBm) is divided in two branches. The lower one carries the continuous probe wave through an optical isolator and a variable attenuator is used to adjust its power just before entering the fiber under test (FUT). In order to avoid deceptive Brillouin frequency determination due to non local effects, the probe power entering a fiber longer than 22 km should not exceed –14 dBm, in the worst case scenario for a standard fiber, as explained in [16]. However, assuming that the fiber used in our experiments is not completely homogeneous all along its length, the probe power can be slightly higher. The upper branch amplifies the laser output up to 21 dBm and launches it into a Brillouin generator that consists of a circulator and a 5-km length of HNF. This device generates a new optical signal that is downshifted in optical frequency by the Brillouin frequency shift in the HNF. Figure 5 shows the resulting spectrum observed in the optical domain with a BOSA (Brillouin Optical Spectrum Analyzer) [17] and in the electrical domain with an ESA (Electrical Spectrum Analyzer). The optical spectrum depicts the downconverted optical wave as well as the Rayleigh scattering to the laser. In the electrical domain the beat of both waves is obtained at the Brillouin frequency shift in the HNF. Note that in a practical application the temperature of the HNF should be stabilized to maintain a constant Brillouin frequency shift. During the experiments, the fiber was kept in a temperature isolated expanded polystyrene box. In addition, notice that the EDFA before the circulator would not be needed if a high power CW DFB laser were deployed, reducing the setup costs

**Figure 6.**
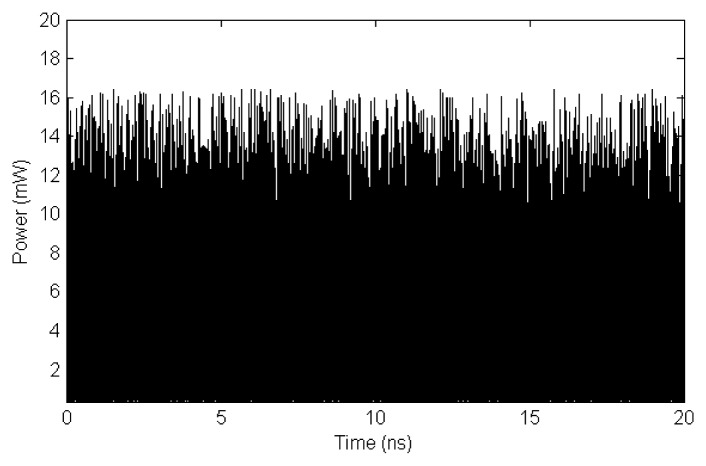
Temporal evolution of the power of the Stokes signal generated in the HNF.

The first downshifted photons are generated inside the HNF through the spontaneous Brillouin scattering process and then, they are amplified by SBS all along its way back to the circulator. This implies that the Stokes signal observed at the exit of the circulator has been generated from thermally excited phonons and therefore, it has a noisy intensity distribution as observed in Figure 6. This distribution was studied by R.W. Boyd *et al.* [18] assuming that the thermal excitation of the phonons in the fiber can be treated as a Langevin noise source. From their theoretical analysis they conclude that the Stokes wave intensity depends on the single-pass gain (G), described by the expression:

(1) $$\text{G}={\text{g}}_{\text{B}}\text{I}\left(0\right)\text{L}$$

where L is the length of the fiber, I(0) is the intensity of the pump wave at the entrance of the fiber and g_B_ is the Brillouin gain coefficient of the fiber. The bigger the value of G, the higher would be the intensity of the Stokes wave and the smaller its fluctuations due to the saturation effect in pump depletion regime. That is why the EDFA located before the HNF should be set at its maximum output power (21 dBm). Also the temporal scale of the fluctuations increases with G. The power fluctuations can be characterized by it variance, defined as:

(2) $$\text{Var}\left(P\right)=\langle P^{2}\rangle -{\langle P\rangle }^{2}$$

When we are not in pump depletion regime and therefore G is small, the normalized variance ${\left(\text{Var}\left(\text{P}\right)/<P{>}^{2}\right)}^{\frac{1}{2}}$ will be equal to 1, since the Stokes power comes from the linear amplification of the waves scattered by thermally generated phonons, having the characteristics of thermal light. In pump depletion regime, this value starts decreasing due to the saturation of the amplification process. In our system, the normalized variance of the Stokes power measured at port 3 of the circulator has a value of 0.7 ensuring that we are in pump depletion regime.

**Figure 7.**
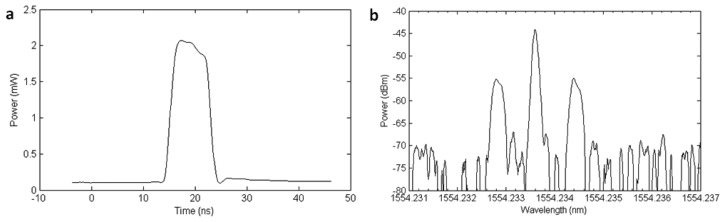
(**a**) Optical pulses generated in the system; (**b**) Optical spectrum at the exit of the EOM.

The Brillouin frequency shift for the HNF fiber is 9.992 GHz, while the standard singlemode fibers used to monitor the structures have *ν_B_* around 10.8 GHz. This frequency shifted signal comes out from the circulator and is RF modulated in the MHz range by an electro-optical modulator (EOM) in order to obtain the final optical frequency tuning and shifting [15]. Before entering the EOM, the signal is cleaned up by means of a 1 nm bandwidth tunable filter, removing the first EDFA's amplified spontaneous emission (ASE). A polarization controller is also placed before the entrance to the EOM, in order to adjust the polarization state of the incoming waves to optimize the carrier component suppression. Pulsing of the signal is done in the RF domain by means of an RF switch and a pulse generator [19]. Figure 7 shows the shape of the pulses as well as the optical spectrum at the exit of the EOM. The amplitude of the pulses is adjusted and its polarization scrambled in order to avoid modulation instability [20] and polarization dependence respectively in our measurements. The optical power of the pulses right after the EOM is 0.1 dBm, so they need to be amplified by an EDFA before entering the fiber under test (FUT).

**Figure 8.**
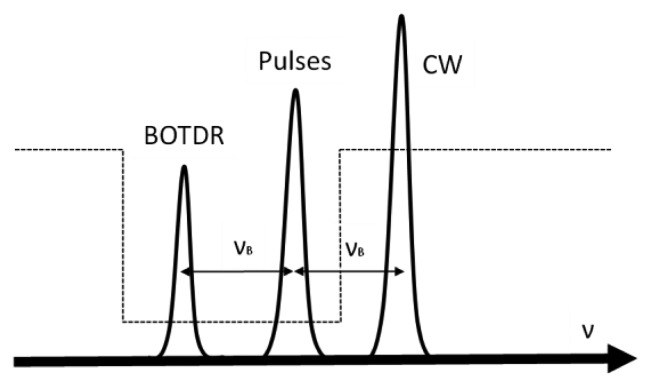
BOTDA signal filtering by means of a transmissive FBG represented by the dashed line.

After interaction in the FUT, the BOTDA signal comes out from port 3 of the second circulator shown in the scheme, and it is filtered out and boosted up prior to be captured by a low noise photodetector. The filtering is carried out by means of a transmissive 0.3 nm wide FBG, cutting off the Rayleigh scattering of the pulses as well as the BOTDR (Brillouin Optical Time Domain Reflectometry) signal generated along the fiber due to spontaneous Brillouin scattering, as shown in Figure 8. The filtered signal is amplified by an EDFA whose output signal is cleaned up with a tunable bandpass filter as done in the upper branch. Then, it is analyzed in an oscilloscope. The oscilloscope and the RF generator are computer controlled in order to sweep the modulation frequency around the Brillouin gain curve of the FUT. This configuration allows us to use a low-speed EOM with relatively low extinction ratio (ER) as well as a low frequency RF generator (in the MHz range), avoiding the use of costly wide-band specialty high-ER EOM and microwave synthesized generators

## 3. Results

### 3.1. Simplified BOTDA Measurements

**Figure 9.**
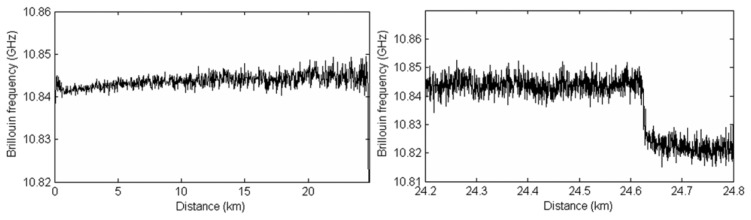
(**a**) Brillouin frequency shift along the sensing channel; (**b**) Detailed view of the 180 m long section at the end of the sensing channel.

The operation of the BOTDA system was initially tested for a 25 km long fiber channel. The result of the temperature measurements along the fiber are shown in Figure 9. The pulses were 10 ns long, achieving therefore a spatial resolution of 1 m. A minimum of 5 ns pulses can be achieved by the RF switch used. The power of these pulses was adjusted to be 9.5 dBm at the entrance of the fiber, avoiding the effect of modulation instability. The CW power was limited to –13 dBm to avoid non local effects. A 180 m long section of a fiber with a different *ν_B_* was added at the end of the channel. Figure 9b shows a zoom of the last part of the fiber channel, were it can be observed that the added section has a Brillouin frequency shift approximately 25 MHz lower than the previous fiber. The standard deviation for the Brillouin frequency shift at the end of the 25 km fiber was 1.60 MHz, resulting in an estimated temperature measurement precision (2σ) of around 3 °C using 2,048 averages.

### 3.2 Sensor Network Validation

In order to check the performance of the full sensor network, proof-of-concept experiments were carried out in our laboratory. 400 mW of Raman pump were launched into the fiber (200 mW on each branch) to feed up the photoelectric cells that act as a power supply for the optical switches. The Raman signal could also contribute to compensate the optical losses of the two 10 km sensing lines due to the induced distributed Raman gain along the fiber; however, given the deployed power, the Raman gain was negligible in the current setup. Another optical signal was needed to remotely control the switch position. We used the output of a tunable laser source, tuned at 1,546 nm, but any stable light source is appropriate as long as the receiver gets at least –28 dBm at any wavelength between 1,310 and 1,550 nm. The power of the continuous probe wave was –8 dBm, and the power of the pump pulses was 24.4 dBm at the entrance of the network. The pulses were 20 ns long, so the measurements have 2 m as spatial resolution.

**Figure 10.**
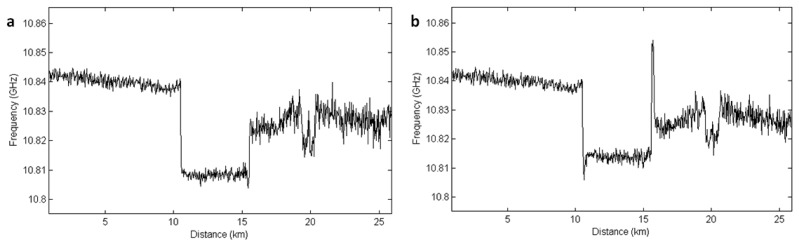
Brillouin frequency shift along the sensing channel 1 (**a**) and the sensing channel 2 (**b**).

**Figure 11.**
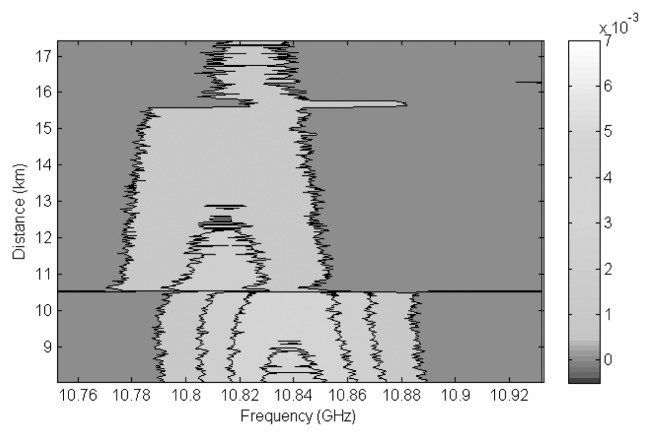
Brillouin gain spectra along the sensing channel focused on the 5 km section with 180 m heated to 60 °C.

In the section to be monitored, between the switches, we placed 2 fiber spools of 5 km SMF. At the end of one of them, we added 180 m of the same fiber. The temperature of this added section was kept constant at 60 °C. Figure 10 shows the value of *ν_B_* for each sensed branch. We can clearly distinguish the two 10 km long sections before and after the 5 km spools. Note that de second section of 10 km is in fact the ensemble of two spools of 6 and 4 km, respectively, spliced together. We observe in Figure 10b the results obtained for the spool to which we added 180 m of heated SMF. We clearly see that *ν_B_* for this region has moved up to 10.852 GHz, 39 MHz higher than *ν_B_* for the previous 5 km, kept at room temperature. This agrees with the well known *ν_B_* shift ratio in SMF of 1.15 MHz/°C [21]. The difference observed between the two measurements in the 5 km section (*ν_B_* varies from 10.808 to 10.813 MHz) is due to slight differences in the two spools, *i.e.*, different winding tension. The measured temperature precision in the 5 km was estimated to be 2.8 °C. In Figure 11 we observe the Brillouin spectra along the 5 km + 180 m section. It can be seen that the Brillouin gain curve shifts to lower frequencies when entering the 5 km section from the 10 km SMF channel, and moves up in frequency for the heated 180 m long section.

## 4. Conclusions

A new optical sensor network based on BOTDA technology has been presented and experimentally validated. A fast remotely and optically controlled power by light optical switch has been used to select the structure to be sensed 10 km away from the interrogation unit. We performed temperature distributed measurements along 25 km where 2 different sections of 5 km placed in the middle were measured sequentially with 2 m resolution. The BOTDA unit employed to interrogate the structures uses a simplified configuration that allows avoiding wide-band high-ER specialty EOMs and high frequency microwave synthesized generators. It is also possible to use de-noising techniques for Raman-assisted applications as proposed in [22].
